# Genetic and other factors determining mannose-binding lectin levels in American Indians: the Strong Heart Study

**DOI:** 10.1186/1471-2350-10-5

**Published:** 2009-01-22

**Authors:** Lyle G Best, Robert E Ferrell, Susan DeCroo, Kari E North, Jean W MacCluer, Ying Zhang, Elisa T Lee, Barbara V Howard, Jason Umans, Vittorio Palmieri, Peter Garred

**Affiliations:** 1 Missouri Breaks Industries Research Inc, Timber Lake, SD, USA; 2 University of Pittsburgh, Pittsburgh, PA, USA; 3 University of North Carolina, Chapel Hill, NC, USA; 4 Southwest Foundation for Biomedical Research, San Antonio, TX, USA; 5 University of Oklahoma Health Sciences Center, Oklahoma City, OK, USA; 6 Medstar Research Institute, Washington, DC, USA; 7 Cornell Medical Center, New York, NY, USA; 8 Rigshositalet, University of Copenhagen, Copenhagen, Denmark

## Abstract

**Background:**

Mannose-binding lectin (MBL) forms an integral part of the innate immune system. Persistent, subclinical infections and chronic inflammatory states are hypothesized to contribute to the pathogenesis of atherosclerosis. MBL gene (*MBL2*) variants with between 12 to 25% allele frequency in Caucasian and other populations, result in markedly reduced expression of functional protein. Prospective epidemiologic studies, including a nested, case-control study from the present population, have demonstrated the ability of *MBL2 *genotypes to predict complications of atherosclerosis,. The genetic control of *MBL2 *expression is complex and genetic background effects in specific populations are largely unknown.

**Methods:**

The Strong Heart Study is a longitudinal, cohort study of cardiovascular disease among American Indians. A subset of individuals genotyped for the above mentioned case-control study were selected for analysis of circulating MBL levels by double sandwich ELISA method. Mean MBL levels were compared between genotypic groups and multivariate regression was used to determine other independent factors influencing *MBL2 *expression.

**Results:**

Our results confirm the effects of variant structural (B, C, and D) and promoter (H and Y) alleles that have been seen in other populations. In addition, MBL levels were found to be positively associated with male gender and hemoglobin A1c levels, but inversely related to triglyceride levels. Correlation was not found between MBL and other markers of inflammation.

**Conclusion:**

New data is presented concerning the effects of known genetic variants on MBL levels in an American Indian population, as well as the relationship of *MBL2 *expression to clinical and environmental factors, including inflammatory markers.

## Background

Early innate human defenses against microbial invaders include mannose-binding lectin (MBL), a serum protein, which acts as a microbial opsonin and initiates activation of the so-called lectin complement activation pathway. Deficiencies in innate immunity have been hypothesized to allow chronic inflammatory states that may contribute to the pathogenesis of atherosclerosis [1]. Common variations in the MBL gene (*MBL2*), between 12 to 25% allele frequencies in Caucasian and other populations [2], result in markedly **lower **levels of circulating MBL [3] and are associated with both an increased susceptibility to infections [4] and cardiovascular disease in many [5-8], but not all studies [9]. An independent association between variant *MBL2 *genotypes and coronary artery disease (CAD) was previously confirmed in the American Indian, Strong Heart Study (SHS) cohort (OR = 3.2, 95% CI 1.5–7.0, p = 0.004, adjusted for 11 CAD covariates) [10]. On the other hand, genetypes resulting in **high **levels of MBL may be involved in complications related to cardiovascular disease (CVD). Thus, the role of MBL in cardiovascular disease appears to be a double-edged sword, due to hitherto only partially resolved mechanisms [11-13].

Although considerable effort has focused on the genotypic determination of MBL levels, our understanding of the role of various promoters and structural genetic variations remains incomplete. New promoter variants continue to be identified [14,15] and genetic background effects in specific populations are largely unknown.

MBL is also known to increase during an acute-phase reaction [16]. Samples collected during follow-up of a subset of the SHS cohort provide a valuable opportunity to test the correlation of both genotype and environmental exposures (e.g. incident CAD and inflammation, as measured by C-reactive protein (CRP) or fibrinogen) with MBL levels in this population.

## Methods

The previously mentioned SHS case/control study matched cases and controls on age, gender and recruitment center (Arizona (AZ), Dakotas (DK) and Oklahoma (OK)). From this group a subset was chosen to include 186 of the controls without regard to genotype and an additional 51 cases limited to those known to be homozygous for high expressing "HYA" alleles.

Ascertainment of fatal and nonfatal cardiovascular events occurring between examinations was accomplished by medical record review and/or yearly participant contact [17]. Cases were defined by evidence of definite myocardial infarction (MI), definite coronary artery disease (CAD) without MI, definite evidence of MI by Minnesota ECG coding [17], or mortality codes indicating either definite MI, sudden death due to CAD, or definite coronary heart disease occurring between 5/18/89 and 12/31/99 [18]. Participants with only a diagnosis of possible CAD, "other CAD", stroke, congestive heart failure, or peripheral vascular disease were excluded. Controls were those individuals without any of the above diagnoses. MBL levels, *MBL2 *genotypes and other covariates were determined from both of these groups. American Diabetes Association criteria [19] were used to classify participant diabetic status in table 1. Participants were considered hypertensive if they were taking anti-hypertensive medications, had a systolic blood pressure greater than 140 mm Hg, or a diastolic blood pressure greater than 90 mm Hg.

**Table 1:** Characteristics of cases and controls analyzed.

Characteristic	Cases N = 51	Controls N = 186
Gender (% Female)	49	52

Age (years)	60.1	58.7

Percentage of participants reporting 100% AI heritage	78	68

Median percent AI heritage among those with less than 100%	65.6	50

Diabetes Mellitus (%)	64.7	39.8

Hypertension (%)	27.5	22.6

Smoking, current (%)	37.3	25.3

Alcohol, current use (%)	41	36

Weight (Kg)	82.9	84.2

BMI (Kg/m^2^)	29.4	30.4

Hgb A1c (%)	8.2	6.4

LDL Cholesterol (mg/dl)	130.2	119.1

Triglycerides (mg/dl)	198.1	125.5

CRP (mg/dl)	2.99	3.45

Fibrinogen (mg/dl)	319.8	304.7

PAI-1 (mg/dl)	43.3	46.8

Determination of MBL levels was done at Rigshositalet in Copenhagen, Denmark, using a double sandwich ELISA method as previously described [20].

The *MBL2 *gene was assessed for the presence of the B (G54D), C (G57E) or D (R52C) structural variations, and two promoter polymorphisms, one a G/C transition at -550 bp (the H/L alleles) [21] and the other, a G/C transition at -221 (X/Y alleles). These structural variations have typically been labeled "O" alleles in contrast to the most common "A" allele. Genotypes were determined by the oligonucleotide ligation assay as described by Nickerson and colleagues [22]. Quality control, duplicate, genotyping was performed by direct DNA sequencing. The structural variations were assumed to occur on opposing chromosomes. A number of promoter variants and structural alleles have been found to be in complete linkage disequilibrium and genotypes were checked against these established relationships. [23]. Genotyping was conducted at the University of Pittsburgh, Pittsburgh, Pennsylvania.

Composite categories of genotypes predicted to result in reduced expression of *MBL2 *similar to previous reports [3], primarily A_A, A_O, O_O, YA_O, XA_O, HA_O, LA_O, HYA_O, LYA_O, LXA_O, along with selected specific haplotypes of adequate prevalence, were used to guide analysis of genotypic effects on MBL levels. More inclusive categories used in the previous SHS *MBL2 *analysis [10] were also considered. Low expression of *MBL2 *(LOW_1) was assumed from the presence of an O_O or LA_O genotype. The LOW_2 category consisted of either O_O, LA_O or LA_LA genotypes. These risk categories were compared with reference groups ALL_1 or ALL_2, consisting of all genotypes not included in either LOW_1 or LOW_2 respectively; or the HIGH category (either HA_HA or HA_LA). There is ample documentation of the biologic effect of these various genotypes on basal MBL levels [23], although unidentified background genetic influences in unique populations are of interest.

The Chi square statistic was used to evaluate proportions between groups. Multivariate regression models and comparison of means (t test of independent samples) were used to understand the contribution of the various factors to *MBL2 *expression using SPSS version 10.1.0 software. The distributions of all subgroups in table 2 were examined and no deviation from the normal distribution was detected. A nominal p value of 0.05 was used to determine statistical significance and the Bonferroni correction was applied in situations of multiple testing.

**Table 2:** MBL levels (μg/L) found in various genotypes and genotypic categories.

Haplotype	N	Min	Max	Mean	SD
Controls					

HYA_HYA	76	128	3920	1863.2	854.95

HYA_LYA	34	400	3296	1422.6	826.14

HYA_LXA	21	223	2848	1030.8	709.6

LYA_LYA	2	1040	3024	2032.0	1402.9

LYA_LXA	1			1376.0	

LXA_LXA	1			3616.0	

HYA_B	27	0	768	168.9	150.7

HYA_C	1			273.0	

HYA_D	7	157	1840	696.29	585.9

LYA_B	8	24	233	115.25	84.1

LYA_D	1			448.0	

LXA_B	1			0.0	

B_B	6	0	0	0.0	

YA_O	44	0	1840	251.7	321.9

XA_O	1			0.0	

XA_O or O_O	7	0	0	0.0	

HA_HA	76	128	3920	1863.2	854.9

HA_LA	55	223	3296	1273.0	800.3

LA_LA	4	1040	3616	2264.0	1250.6

HA_O	35	0	1840	277.3	351.3

LA_O	10	0	448	137.0	137.0

HIGH	131	128	3920	1615.4	879.3

**LOW_1**	16	0	448	85.6	126.3

**LOW_2**	20	0	3616	521.3	1029.0

A_A	135	128	3920	1634.7	893.0

Not "A_A"	51	0	1840	217.2	311.1

Cases					

HYA_HYA	51	640	5888	1903.6	1043.4

Approval consistent with the Helsinki Declaration was obtained from all relevant Institutional Review Boards and tribes, and all participants gave informed consent.

## Results

Table 1 summarizes the characteristics of both cases and controls. Please note that cases for this study were chosen on the basis of genotype and since cases and controls were initially matched for gender, age, and recruitment center, comparison between aggregated case and control samples should be interpreted with caution.

There were no significant differences in the prevalence of O (15.3%) and H (67.2%) alleles in this control sample, compared with an earlier SHS report [10].

These findings confirm low levels of MBL among individuals with composite genotypic groups (eg O_O, XA_O, LA_O) [2], as well as those previously associated with CAD in this SHS population (bolded) [10], as seen in table 2.

**Figure 1:**
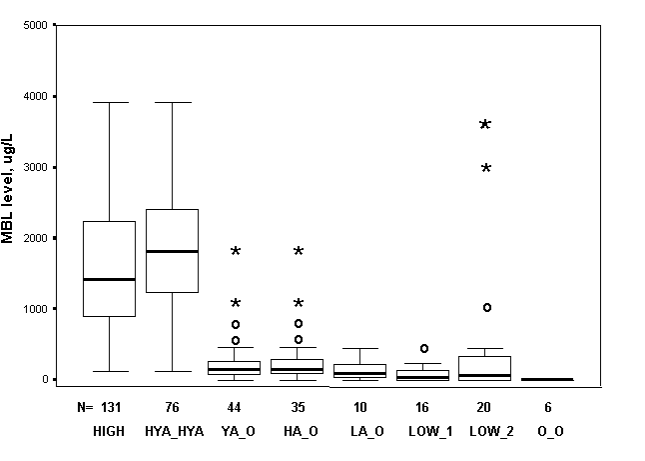
**MBL levels for various genotypes**. Comparison of mean HIGH and HYA_HYA MBL levels with all other groups is significant at p < 0.001. Outliers (between 1.5 and 3 box lengths from upper and lower margin) and extreme values (greater than 3 box lengths from box margin) are denoted by "o" and "*" respectively.

Among controls only, comparing means of composite genotypes showed highly significant differences (p < 0.001 for all comparisons) between either the HYA_HYA or HIGH groups and any of the YA_O, HA_O, LA_O, O_O, LOW1, or LOW2 groups. Figure 1 shows these comparisons in the familiar "box-plot" format. The difference in mean MBL between HYA_HYA and HYA_LYA (unadjusted for multiple testing, p < 0.013), was not significant considering a Bonferroni correction requiring a p < 0.00365 for a total of 14 pair-wise tests. No significant differences were found between MBL means of HYA_LYA and HYA_LXA or HYA_B and LYA_B (although the last category was represented by only 8 individuals); other comparisons attempting to isolate the influence of the H/L or X/Y promoters were not possible due to small numbers of individuals in categories.

Only cases with HYA_HYA genotype were included to allow adequate numbers to compare with controls of identical genotype. If the mean MBL levels for case and control groups of identical genotype are compared (ignoring the previously matched selection of cases and controls on the basis of age, gender and center), then a crude p value of 0.812 is obtained.

Multivariate regression analysis was used to investigate the interplay of various demographic, environmental, genetic factors on expression of *MBL2 *within the control group. A backward selection criterion of F ≥ 0.1 was used and results are summarized in table 3. Male gender, hemoglobin A1c levels and self-reported % American Indian ethnicity are positively correlated with MBL levels; whereas the converse is true of increasing triglyceride levels and the number of B alleles. Covariates not retained in this backward selection included: age, diabetes mellitus, systolic blood pressure, Chlamydia pneumoniae (Cp) antibody titers, percent body fat, and urinary albumin/creatinine ratio. Although MBL is an acute phase reactant, it was not correlated with either inflammatory measures (CRP, PAI-1, fibrinogen, WBC count) or infectious disease exposure (Cp titers).

**Table 3:** Results of multivariate regression analysis on the control group (n = 186).

Factor	beta coefficient	p value
MALE GENDER	.161	.011

% AMERICAN INDIAN ETHNICITY	.216	.001

ln TRIGLYCERIDES	-.159	.013

HEMOGLOBIN A1c	.219	.001

## Discussion

Genetic variation in the *MBL2 *gene has been implicated in important pathophysiologic processes including susceptibility to infections [24-26], chronic inflammatory states and their sequelae [5,6,8,27], and the response to acute pathology, such as ischemic necrosis [28]. Further down the pathogenetic chain of events, circulating levels of this protein are also associated with pathology [27,29,30]. It is therefore important to fully understand the genetic and environmental factors governing expression of this protein. Since MBL is a polymeric protein comprised of self-assembled trimeric subunits, dominant-negative structural variants were first recognized to have marked effects on functional MBL levels. Promoter polymorphisms were soon recognized as key determinants of *MBL2 *expression and as recently as 2006, new variants have been identified [3,15,31]. It is likely that additional influences (eg the endocrine milleu [32,33], variation in multimeric assembly [34], and epigenetic effects determined by the "background" population [3] on *MBL2 *expression and function will continue to be uncovered. The worldwide high population prevalence of these variants suggests an apparently ambivalent role of *MBL2 *in a number of pathogenetic and homeostatic processes [35-37].

In this report we have generally confirmed the previously reported influence of the three main structural variants and the two most widely investigated promoter variants (H/L and X/Y). This is of importance due to the demonstrated association between CVD and these variants in this particular, non-Caucasian population. Other studies have reported on the prevalence of *MBL2 *variants in native populations of North America [38]; but have not included information on the expression of *MBL2*, as in the present study. Since MBL levels are higher in those with diabetes (although not seen in this dataset) [27], a correlation with HgbA1c could be expected. The finding of an association with lower levels of triglycerides in adjusted analysis is more difficult to explain, especially since triglyceride levels are often increased in those with diabetes or the metabolic syndrome [39]. Others have also reported increased MBL levels among males [30]; but no previous analyses of triglycerides in relation to MBL could be found in the literature.

Strengths of this study include confirmation of the presumed link between genotype and expressed protein, increasing confidence in a possibly causal association between MBL and cardiovascular disease in this population. These results also extend the previously identified genotype/phenotype correlations to another population with a unique genetic background. A weakness is the previous matching structure of the sample and the need to compare HYA/HYA case genotypes with the same control genotypes. There were an insufficient number of previously matched pairs with this genotype to allow a matched analysis, thus the aggregate comparison between these two groups needs to be interpreted with caution.

## Conclusion

The findings from our study corroborate previous research that has demonstrated the important effects of genetic variants and the association of metabolic abberations with MBL levels. Further understanding of *MBL2 *expression may improve our ability to understand and disrupt the pathogenetic mechanisms involved in cardiovascular disease.

## Competing interests

The authors declare that they have no competing interests.

## Authors' contributions

All authors have read and approved the final manuscript. LB: Originated and conducted the majority of the analysis, drafted the manuscript and is the corresponding author. RF and SD: Supervised and conducted the genotyping. KN, JM, EL, BH, JU and VP: Provided recommendations on analysis and assisted in editing the manuscript. YZ: Provided provided critical statistical supplort and validation. PG: Supervised laboratory determination of MBL levels, as well as providing major contributions in developing the analytic strategy and key editorial recommendations.

## Pre-publication history

The pre-publication history for this paper can be accessed here:


